# Non-invasive monitoring of blood gas-induced changes of myocardial oxygenation using oxygen-sensitive CMR

**DOI:** 10.1186/1532-429X-14-S1-P285

**Published:** 2012-02-01

**Authors:** Dominik P Guensch, Kady Fischer, Jacqueline Flewitt, Janelle Yu, Ryan Lukic, Julian A Friedrich, Matthias G Friedrich

**Affiliations:** 1 Kady Fischer, Stephenson Cardiovascular MR Centre, Calgary, AB, Canada

## Summary

BOLD-CMR was used to assess changes in myocardial oxygenation after volunteers performed controlled hyperventilation or breath holding. Signal intensity after hyperventilation decreased whereas an increase occurred after a breath hold demonstrating that controlled breathing techniques could alter myocardial oxygenation and be identified by BOLD-CMR in healthy volunteers.

## Background

Systemic changes of blood gases (CO_2_, O_2_) affect haemoglobin (Hb) saturation. Blood Oxygen Level-Dependent (BOLD-) CMR can be used to monitor changes of myocardial oxygenation. We hypothesized that oxygen-sensitive CMR detects changes in myocardial tissue oxygenation induced by hyperventilation and apnea.

## Methods

A group of 7 healthy volunteers were instructed to hyperventilate for 1 and 2 minutes followed by a long free breath hold. A second group of 5 aquatic athletes performed a 60s breath hold and a free maximal breath hold. BOLD-sensitive SSFP cines were acquired during breath holds as well as before and after hyperventilation. Changes in signal intensity over the procedures were expressed as % change of the baseline. Capillary blood gases were measured prior to and after the procedures.

## Results

Voluntary breath holds of athletes were significantly longer (105±38s) than those of other volunteers (38±12s). Breath holds lead to a significant increase in signal intensity (*p<0.001), correlated with the length of breath hold (R=0.566, *p=0.018). Capillary pCO_2_ did not change during breath holds, while pO_2_ increased during shorter breath holds of 38s (+8.8 mmHg, *p=0.03) and decreased in long breath holds of 105s (-14.5mmHg, *p=0.03). On the other hand, hyperventilation resulted in a significant decrease of myocardial signal intensity, associated with a decrease of capillary pCO_2_ of 5.9 mmHg during 1 min of hyperventilation (*p<0.001) and 8.7 mmHg during a 2 min hyperventilation period (*p<0.001). Capillary pO_2_ was not altered by hyperventilation.

## Conclusions

Our results demonstrate that BOLD-CMR can identify changes in myocardial oxygenation induced by controlled breathing maneuvers.

## Funding

Husky Energy Program for the Early Detection of Cardiovascular Disease

**Figure 1:**
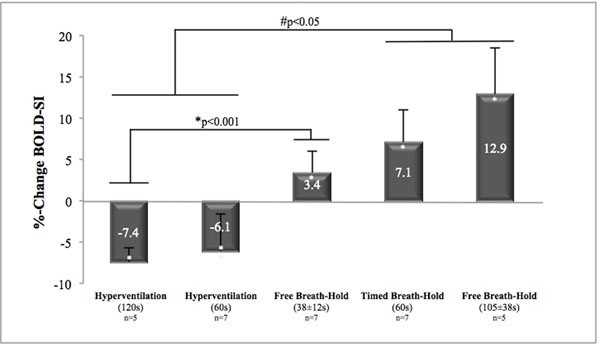
The %-change in SI was significant after hyperventilation or a breath hold (*p<0.001) as the %-change in SI was significant between groups undergoing hyperventilation or a breath hold (#p<0.05).

